# Long-Range Imaging of Alpha Emitters Using Radioluminescence in Open Environments: Daytime and Night-Time Applications

**DOI:** 10.3390/s24165345

**Published:** 2024-08-18

**Authors:** Lingteng Kong, Thomas Bligh Scott, John Charles Clifford Day, David Andrew Megson-Smith

**Affiliations:** HH Wills Physics Laboratory, Interface Analysis Centre, School of Physics, University of Bristol, Tyndall Avenue, Bristol BS8 1TL, UK; t.b.scott@bristol.ac.uk (T.B.S.); john.day@bristol.ac.uk (J.C.C.D.); david.megson-smith@bristol.ac.uk (D.A.M.-S.)

**Keywords:** alpha radiation detection, radioluminescence, alpha fluorescence, long-distance monitoring, solar-blind detector

## Abstract

Alpha emitters like plutonium pose severe health risks when ingested, damaging DNA and potentially causing cancer. Traditional detection methods require proximity within millimeters of the contamination source, presenting safety risks and operational inefficiencies. Long-range detection through alpha radioluminescence (RL) offers a promising alternative. However, most of the previous experiments have been carried out under controlled conditions that preclude the overwhelming effect of ambient light. This study demonstrates the successful detection of a 3 MBq alpha emitter in an open environment using a compact alpha camera. This camera incorporates a deep-cooled CCD and a low f-number lens system designed to minimize the blue shift effects of filters. Night-time imaging was achieved with a dual-filter system using a sandwich filter assembly centered at 337 nm and 343 nm for capturing alpha RL and subtracting background light, respectively. At night, the alpha source was detected from 1 m away within one minute, and the lowest detection limit can be calculated as 75 kBq. The system was also evaluated under simulated urban lighting conditions. For daytime imaging, a stack of tilted 276 nm short pass filters minimized sunlight interference, enabling the detection of the alpha source at 70 cm within 10 min under indirect sunlight. This research highlights the viability of long-range optical detection of alpha emitters for environmental monitoring in real-world settings.

## 1. Introduction

Alpha particles, consisting of two protons and two neutrons, are identical to the nucleus of a helium-4 atom. They are typically emitted through the alpha decay of heavy nuclei, where an unstable atom releases an alpha particle to transform into a different element. The travel range of alpha particles in air is generally limited to several centimeters, depending on their energy, which makes detection challenging [1]. The ingestion of alpha emitters can cause severe DNA damage due to the high linear energy transfer (LET) characteristic of alpha particles. Alpha radiation deposits significant energy over a short track length, causing dense ionization along its path and substantial biological damage [2]. Additionally, alpha radiation can have a broader biological impact through the ’bystander effect’, where non-irradiated cells near irradiated ones also exhibit damage [3]. This was tragically highlighted by the poisoning of Alexander Litvinenko with polonium-210 [4]. Consequently, the effective detection of alpha particles is crucial for protecting public health and the environment from the dangers of alpha radiation.

Current commercial handheld alpha radiation monitors, such as Geiger–Mueller counters and scintillator detectors, require direct proximity to the radioactive source for accurate measurement, typically within millimeters of the contamination area. This process usually requires meticulous and repeated scanning of an area to ensure no contamination is missed, making the detection process labor-intensive and time-consuming. Such procedures not only increase the risk of contamination and dose uptake for workers but also escalate the costs associated with decontaminating or replacing contaminated equipment. For instance, following the assassination of Alexander Litvinenko, the manual use of handheld alpha scanners was necessary to evaluate the spread of radioactive materials across more than 40 sites in London, demanding substantial human resources and time.

Although most alpha emitters also emit gamma rays, which can be detected at long distances, the probability of gamma emission is typically low. This lower likelihood can be attributed to the mechanism of alpha decay, specifically the quantum tunneling effect. If an alpha decay is also accompanied by gamma emission, the kinetic energy of the alpha particle is reduced by the energy of the emitted gamma. Consequently, this increases the thickness of the potential barrier the alpha particle must overcome to exit the nucleus, making its escape even more challenging [5]. For example, Polonium-210 is a pure alpha emitter. Other isotopes such as Plutonium-239, Plutonium-238, and Uranium-238 predominantly decay through alpha emission, with more than 99% of their decays proceeding via this mode, making these isotopes difficult to detect with gamma detectors. In the context of nuclear waste, minor alpha emitters that exhibit high rates of gamma emission, such as Americium-241, which emits a main gamma line of 59.54 keV with a probability of 35.64% [6], also pose detection challenges. Detecting such materials using gamma detectors is difficult because the low-energy gamma emissions are easily overwhelmed by the high gamma background commonly encountered in practical applications, due to the high penetrating power of gamma rays.

Recent advancements in the long-range detection of alpha emitters through radioluminescence (RL) have shown great promise for overcoming the limitations of close-range alpha radiation monitoring [7,8,9,10,11,12,13,14,15,16,17,18,19,20]. This technique leverages the ionization of air molecules by alpha particles. The ionization process releases secondary electrons that excite surrounding nitrogen molecules, emitting photons as they return to their ground state. These photons, primarily in the 280–440 nm range, can travel hundreds of kilometers, detectable by optical instruments like photomultiplier tubes (PMTs) and charge-coupled devices (CCDs) [19,21,22]. The main emission peak at 337.1 nm accounts for 25.7% of total emission [22].

However, the primary challenge in detecting alpha RL is the weak emission intensity. A single 5 MeV alpha particle typically generates only about 100 photons in air, which is over $10^{10}$ times lower than that by ambient light sources [13,23]. Therefore, most of the previous attempts for detecting alpha RL were carried under dark or specific light conditions without a UV background.

For an imaging detector such as a CCD, which is usually sensitive to visible and infrared light, the use of UV-transmissive filters that also block a broad range of visible and infrared light is necessary to reduce ambient background and thereby enable alpha imaging. For environments without a UV background, like indoor LED lighting, the 337.1 nm emission can be effectively used to detect alpha sources. A novel ’sandwich’ filter structure, incorporating an absorptive filter between two interference filters centered at 337 nm, has been developed to minimize multi-reflection between interference filters and enhance blocking efficacy. This setup has enabled the successful imaging of a 29 kBq alpha source from a distance of 3 m within 10 min. It has also proven highly effective within the confines of a glovebox with an acrylic window, which filters most of the ambient UV background outside the glovebox [23].

However, for outdoor imaging, the UV background cannot be ignored. Using only the 337 nm filter system is insufficient to effectively block all ambient background light. Even at night, the moonlight, city light, or astronomical twilight all have spectral components at 337 nm. Daytime imaging presents additional challenges due to the overwhelming UV constituents of sunlight. The UVC region (200 to 280 nm) is largely free from solar interference due to the atmospheric absorption of short-wavelength UV light by oxygen and ozone. This offers a ’solar-blind’ window for outdoor alpha RL detection. However, RL emissions in this spectrum are weak, constituting 0.42% of total emissions [24], which is 61 times lower than the main emission at 337 nm. These UVC emissions are predominantly generated by trace amounts of nitric oxide (NO) in the air [19,25]. Unfortunately, the effective sandwich filter configuration is infeasible for UVC imaging as there are no commercially available absorptive filters with sufficient transmission in the UVC range and broadband blocking across the visible and infrared regimes.

Recent experiments have explored using PMTs to detect the UVC emissions of alpha RL [12,13]. However, these experiments are still under controlled indoor lighting conditions, limiting their application in real-world settings. Additionally, PMTs require a narrow field of view and a scanning mechanism to locate alpha sources over large areas, processes which can be labor-intensive and time-consuming.

Therefore, the primary objective of this research is to develop an imaging system capable of accurately mapping the distribution of alpha emitters from a distance, eliminating the need for close contact or extensive scanning processes. This system is designed to perform effectively in open environments, including under sunlight, thereby enhancing the efficiency of alpha radiation surveys and significantly reducing health risks to workers. This technology aims to enhance nuclear safety and facilitate faster decommissioning processes. Additionally, it will allow for a more effective assessment of nuclear waste packages from a safe distance and enable real-time monitoring of inventories within gloveboxes, contributing substantially to safer and more efficient operational practices in radioactive environments.

## 2. Materials and Methods

### 2.1. Alpha Sources

For the experiments, an Americium-241 (Am-241) alpha source with an activity of 3 MBq and an active area of 12.5 mm diameter was employed. Am-241 typically emits alpha particles with an energy of 5.5 MeV. In this experimental setup, the alpha source was sealed beneath a thin gold film which slightly reduced the alpha energy to 4.7 MeV.

**Figure 1:**
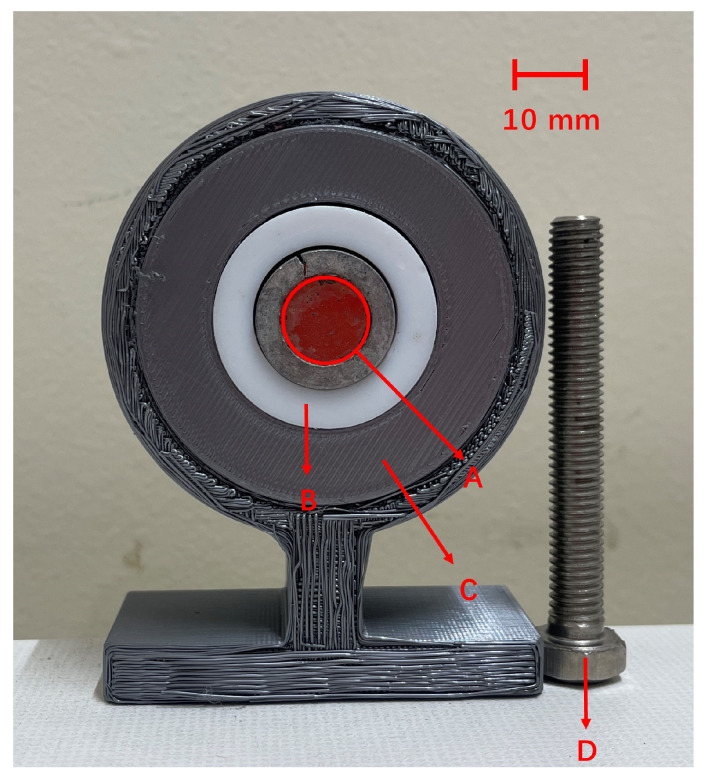
Detailed view of the alpha source setup: (**A**) active area, (**B**) plastic ring, (**C**) 3D-printed polylactide stand, and (**D**) control surface bolt.

The alpha source includes a white polypropylene plastic sealing ring around the active area. The entire source was mounted on a stand fabricated from polylactide using a 3D printer. Adjacent to the alpha source, a metal bolt was placed to act as a control surface. The configuration of the setup is depicted in Figure 1.

### 2.2. Detection System

#### Camera

The detector was a deep-cooled iKon-M 934 BU2 (Oxford Instruments Andor, Belfast, UK) CCD. It originally has 1024×1024 pixels. In order to reduce the read noise per pixel, during the experiment, a 8×8 pixel binning was used. Therefore, the resultant resolution was 128×128 pixels. During the experiment, the camera was cooled to −90 degree. The quantum efficiency (QE), which is the measure of the effectiveness of an imaging device to convert incident photons into electrons, was around 60% in 200–400 nm.

### 2.3. Filter Selection

**Figure 2:**
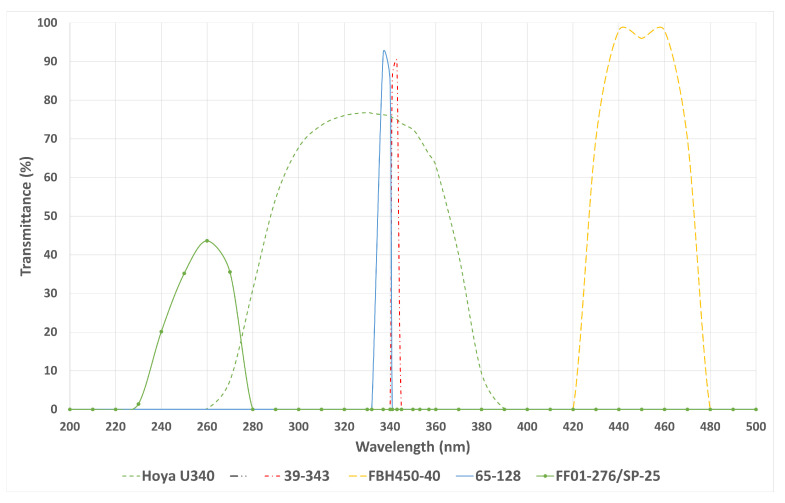
Graph showing the transmission versus wavelength for each filter used in the experiment.

A diverse array of optical filters was selected to optimize detection capabilities and control environmental interference during the experiment. The details of each filter are provided in the Table 1:

Figure 2 illustrates the transmission characteristics of these filters across their respective wavelength ranges.

#### 2.3.1. Filter System for Night-Time Imaging

For night-time imaging, where the ambient UV background is significantly reduced, the 337 nm center wavelength (CWL) filter (65-128) was employed to capture the primary emission of alpha RL. Unlike the distinct peaks characteristic of alpha RL, the UV background at night, such as moonlight or reflections from city lights and sunlight, typically exhibits a continuous broad emission spectrum. To effectively manage any residual UV background, the 343 nm CWL filter (39-343) was utilized for background subtraction. This filter’s transmission wavelength is close to the transmission of 337 nm filter, which means it captures similar UV background. However, it crucially excludes any alpha RL emission peaks, making it highly effective for subtracting background UV influences.

**Figure 3:**
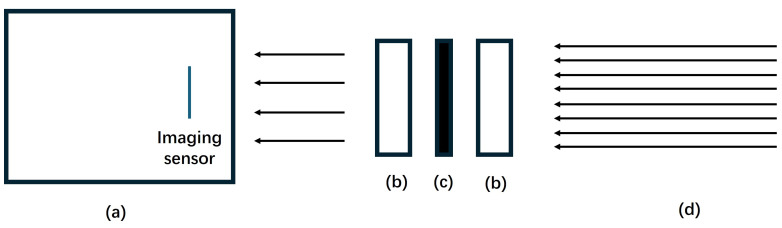
Illustration of the sandwich filter structure: (**a**) CCD and imaging sensor; (**b**) two reflective filters, either 337 nm CWL or 343 nm CWL used in this experiment; (**c**) an absorptive filter, specifically a Hoya U340 used here; (**d**) direction of incoming light.

The filters were arranged in a sandwich structure, as depicted in Figure 3, to enhance the blocking rate of the system. This configuration incorporates an absorptive filter between two reflective (or interference) filters to minimize multi-reflections and enhance image clarity [23]. The transmission of this filter arrangement within the signal wavelength band is 67%.

#### 2.3.2. Filter System for Daytime Imaging

**Figure 4:**
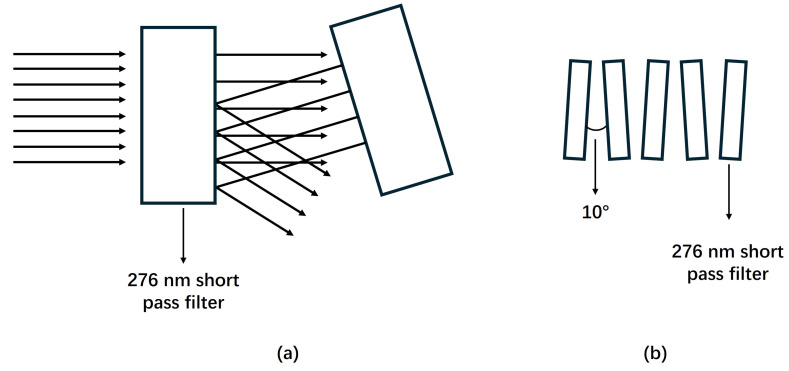
(**a**) By tilting the filters, light that might otherwise reflect between them is directed off-path. After a few reflections, this stray light is absorbed by the lens tube, thereby reducing the light-bunching effect; (**b**) Arrangement of the stacked five 276 nm short pass filters for sunlight detection.

During daytime, imaging under sunlight poses a challenge due to an overwhelming UV background when using a 337 nm filter. Here, 276 nm short pass filters (FF01-276/SP-25) were utilized to reject sunlight effectively. However, a single 276 nm filter is insufficient to completely block sunlight. To overcome this, filters were stacked, and each was tilted using a 3D-printed spacer with a 10 degree angle. This setup reduces multi-reflections by redirecting stray light off-path and into the lens tube after a few reflections, thereby improving filter effectiveness. A sandwich structure was not used here because an absorptive filter with good UVC transmission and high blacking rate in the visible and infrared region was not found. A total of five 276 nm filters were stacked to ensure thorough sunlight exclusion. Each filter had a transmission of around 50% in the UVC signal band. Therefore, the cumulative transmission of the stack of five filters was calculated as ${\left(50\%\right)}^{5}=3.1\%$. A diagram demonstrating the theory behind the filter tilting and the specific arrangement used is shown in Figure 4.

#### 2.3.3. Optical Configuration

For the optical setup of this experiment, a triplet lens system was designed. The specifications of each lens are summarized in Table 2.

**Figure 5:**
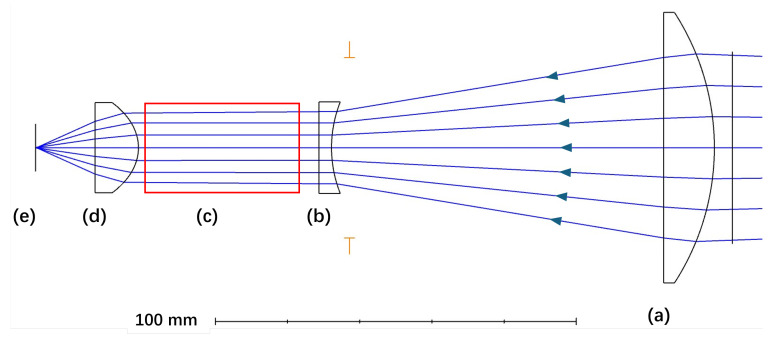
Configuration of the lens system: (**a**) LA4372-UV, (**b**) LC4513-UV, (**c**) space allocated for filter arrangement as depicted in Figure 3 and Figure 4, (**d**) 21-912, and (**e**) the image sensor of the camera.

Each lens was constructed from UV fused silica to maximize the transmission of the alpha RL signal. An anti-reflection coating was applied to each lens to further enhance transmission efficiency and image quality. To further improve image quality, an aspheric lens (21-912, Edmund Optics, York, UK) was selected as the imaging lens. The entire lens assembly was housed in standard lens tubes provided by Thorlabs Ltd., Lancaster, UK. The assembly configuration, including the position of lenses and filters, is detailed in Figure 5.

A key design criterion was to ensure that incoming light rays in the space allocated for the filter arrangement (see Figure 5c) were approximately normal to the filter surfaces. This orientation minimizes the risk of blue shift, which occurs when the center wavelength of a filter shifts toward shorter (blue) wavelengths as the angle of incidence increases, potentially impacting the fidelity of wavelength detection.

The effective focal length (EFL) is estimated using the lens combination formula [26]:

(1) $$\frac{1}{\mathrm{EFL}}=\sum_{i=1}^{n}\frac{1}{f_{i}}=\frac{1}{150\;\mathrm{mm}}+\frac{1}{-75\;\mathrm{mm}}+\frac{1}{25\;\mathrm{mm}}$$

After calculating the values in the equation, the EFL was determined to be 30 mm. The aperture *D* was determined from simulations using Zemax 2016 and was found to be 60 mm. Consequently, the f-number was calculated as follows:

(2) $$f-\mathrm{number}=\frac{\mathrm{EFL}}{D}=\frac{30\;\mathrm{mm}}{60\;\mathrm{mm}}=0.5$$

The angular field of view was measured to be 7.2 degrees.

**Figure 6:**
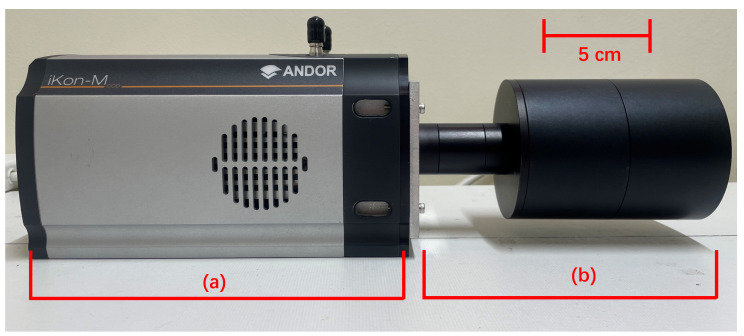
Comprehensive view of the alpha camera setup: (**a**) iKon 934 CCD camera (**b**) Integrated lens and filter system.

The overall assembly of the alpha camera is shown in Figure 6, and the total weight was about 3 kg.

#### 2.3.4. Power Supply

Given the lack of readily available power sources in open outdoor environments, a Jackery Explorer 240 portable power station (Jackery, Oakland, CA, USA) was employed to provide power to the camera system. This power supply offers an operational duration of approximately four hours, ensuring continuous functioning during field experiments.

#### 2.3.5. Artificial Light Source

To simulate the ambient light conditions typical of urban environments during night-time imaging, several artificial light sources were utilized. The specifications of these light sources are summarized in Table 3:

### 2.4. Image Acquisition and Processing

The lens arrangement remained consistent across both daytime and night-time imaging sessions. The primary difference lay in the choice of filters: 337 nm (65–128 filter) and 343 nm (39–343 filter) for night-time and 276 nm short pass (FF01-276/SP-25 filter) for daytime. The detection distance, which is the distance between the front of the camera lens and the alpha source, was set at 1 m for night-time imaging and 70 cm for daytime imaging. This variance is attributable to the uncorrected chromatic aberration in the lens system which affects the focal distance, and therefore, the sample distance needs to be changed for detecting the signal at different wavelengths.

Image exposures were several minutes. Raw images were converted from the proprietary format to CSV files using Andor SOLIS software (version 4.30.30024.0). Given the prolonged exposure times, cosmic rays and gamma rays from the Am-241 source introduced significant noise, appearing as intense peaks in individual pixels. To address this, a median filter with a 3×3 kernel (Python 3.11.9, scipy.ndimage package) was applied to all images.

For night-time imaging, the sandwich filter configuration centered at 337 nm was utilized to capture the main alpha RL emission. Subsequently, the 343 nm centered sandwich filter was used to acquire the background image. The positions of both the camera and the source remained unchanged during filter exchanges. The background image was scaled so that its maximum intensity matched that of the signal image. This scaling was necessary to compensate for the difference in FWHM between the two filters, ensuring consistent background intensity across both images. The alpha RL signal was then isolated by subtracting the 343 nm background image from the 337 nm emission image. During this subtraction, any pixel values resulting in less than zero were set to zero, to avoid negative intensity values that are not physically meaningful.

For daytime imaging, the alpha RL was directly captured using a stack of five tilted 276 nm short pass filters designed to reject sunlight effectively and capture the alpha RL in UVC range. No background subtraction was required for daytime imaging as the indirect sunlight captured did not significantly affect the alpha RL detection.

Post-processing involved representing the RL signal using a colormap, which was then superimposed on the grayscale visible light image (captured using the 450 nm FBH450-40 filter) to accurately depict the location of the alpha source. This visualization was executed using Python 3.11.9 and the matplotlib package.

## 3. Results and Discussion

### 3.1. Imaging of Alpha Source at Night-Time

**Figure 7:**
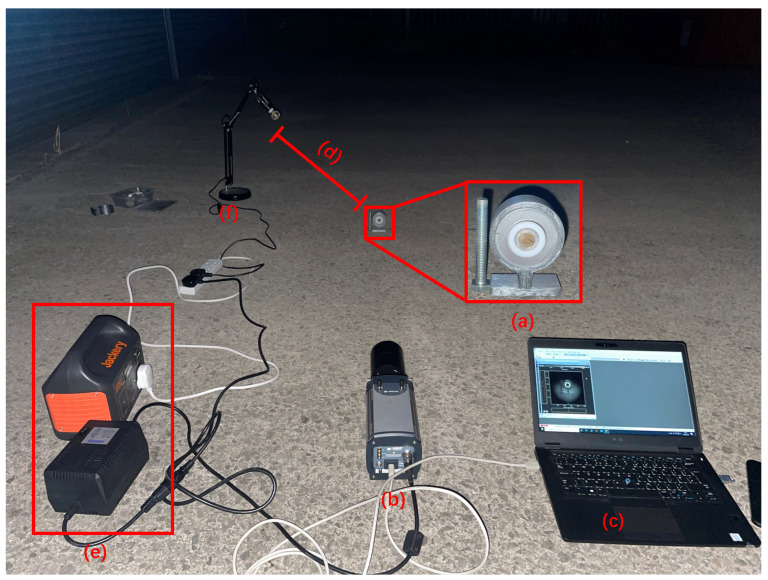
Experimental setup for night-time imaging of alpha RL: (**a**) alpha source, (**b**) alpha camera, (**c**) controlling laptop, (**d**) distance between light bulb and alpha source, (**e**) power bank and supply, (**f**) light lamp simulating ambient light source.

**Figure 8:**
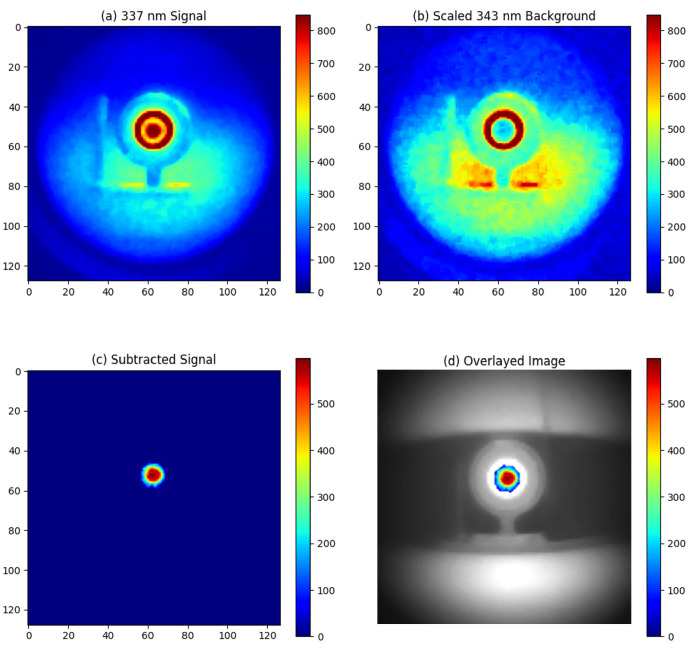
Experimental result for night-time imaging of alpha source at 1 m distance without artificial light source: (**a**) Signal image around 337 nm, (**b**) Background image around 343 nm, (**c**) Signal obtained by subtracting the 343 nm background from the 337 nm emission, (**d**) Overlay of the subtracted signal on a visible light image of the alpha source.

**Figure 9:**
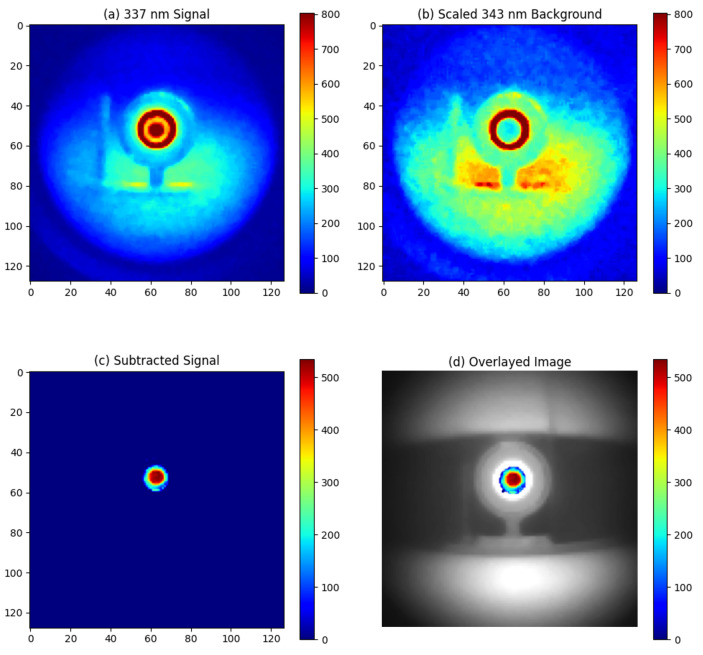
Experimental result for night-time imaging of alpha source at 1 m distance with an LED light source placed 1 m away from the alpha source: (**a**) Signal image around 337 nm, (**b**) Background image around 343 nm, (**c**) Signal obtained by subtracting the 343 nm background from the 337 nm emission, (**d**) Overlay of the subtracted signal on a visible light image of the alpha source.

**Figure 10:**
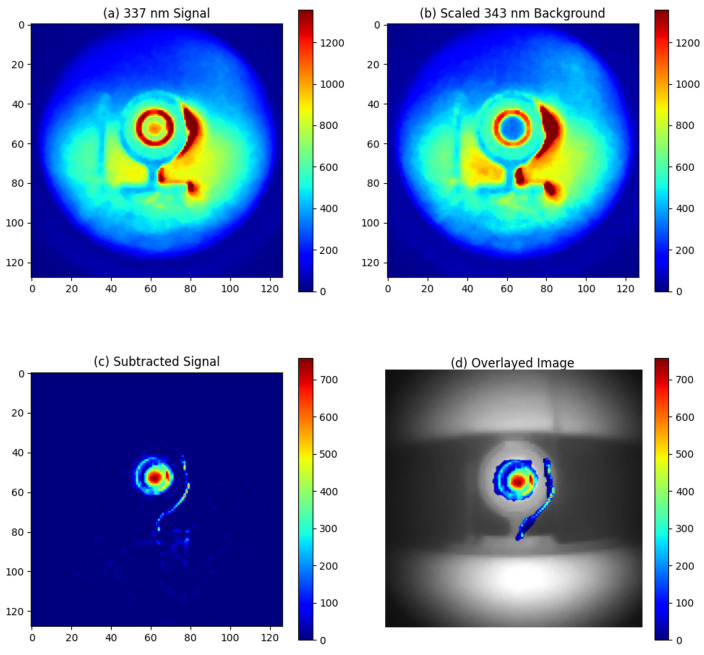
Experimental result for night-time imaging of alpha source at 1 m distance with an incandescent light source placed 8 m away from the alpha source: (**a**) Signal image around 337 nm, (**b**) Background image around 343 nm, (**c**) Signal obtained by subtracting the 343 nm background from the 337 nm emission, (**d**) Overlay of the subtracted signal on a visible light image of the alpha source.

**Figure 11:**
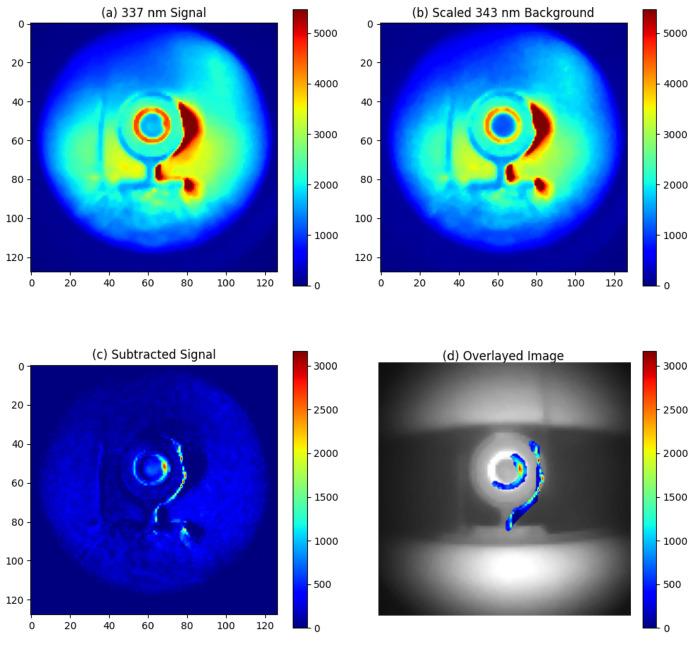
Experimental result for night-time imaging of alpha source at 1 m distance with a fluorescent light source placed 8 m away from the alpha source: (**a**) Signal image around 337 nm, (**b**) Background image around 343 nm, (**c**) Signal obtained by subtracting the 343 nm background from the 337 nm emission, (**d**) Overlay of the subtracted signal on a visible light image of the alpha source.

The night-time experiments were conducted at Fenswood Farm, Bristol, UK, approximately 4.6 miles from the city center to minimize city light interference. Various artificial light sources (LED, fluorescent, and incandescent lamps) summarized in Table 3 were used to simulated different urban lighting conditions. The experiments took place from 00:00 a.m. to 2:00 a.m. on 12 June 2024. During this period, astronomical dusk is not achieved, resulting in UV ambient background from sky illumination (astronomical twilight). The experimental setup is illustrated in Figure 7. The detection distance was 1 m, and the exposure time was 1 min. The results are presented in Figure 8, Figure 9, Figure 10 and Figure 11.

Without artificial lighting, the alpha RL can be observed in Figure 8a. The signal intensity is calculated as the mean pixel value in the area of the alpha source, while the noise is determined by the standard deviation of the pixel values in the background area. The signal-to-noise ratio (SNR) is calculated by dividing the signal by the noise, which results in an SNR of 40. Given that the alpha RL signal is proportional to the activity of the alpha source [13], we can establish the lowest detection limit. By setting the minimum detectable SNR to 1, the lowest activity that can be detected at 1 m with a 1-min exposure is calculated as $\frac{3\;\mathrm{MBq}}{40}=75\;\mathrm{kBq}$.

However, substantial ambient UV light was detected by the 337 nm filter system. For example, the white plastic ring around the active area of the alpha source, which is reflective to UV light, appeared to register high counts, as shown in Figure 8a. By subtracting the 343 nm background image, these ambient light patterns were effectively removed, isolating the alpha source signal as depicted in Figure 8c.

With LED lighting, no additional UV background was observed, demonstrating the effectiveness of the filter system, as illustrated in Figure 9. In contrast, additional UV background was detected with incandescent and fluorescent light sources, as shown in Figure 10 and Figure 11, respectively. The incandescent source, placed 8 m away from the alpha source, allowed for alpha source detection with a SNR of 10. Therefore, the lowest detectable activity under incandescent lighting would be $\frac{3\;\mathrm{MBq}}{10}=300\;\mathrm{kBq}$. However, the fluorescent light source, also placed 8 m from the alpha source, produced an excessive UV background. As seen in Figure 11, this excessive background continued to overshadow the RL signal from the alpha source, even after background subtraction.

### 3.2. Imaging of Alpha Source at Daytime

**Figure 12:**
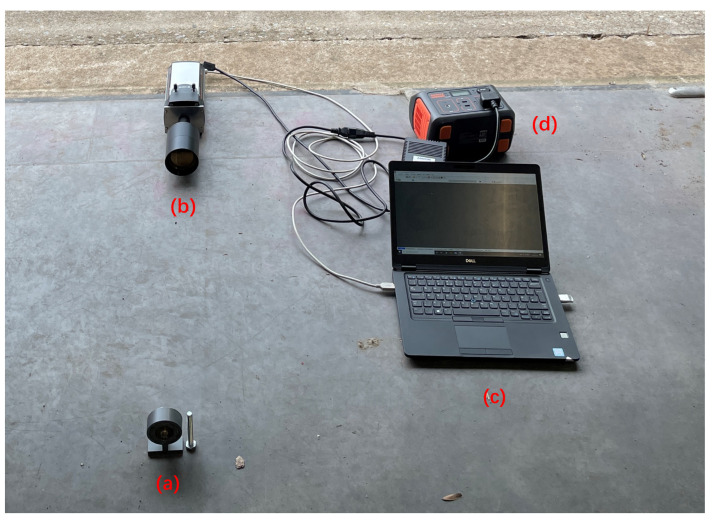
Experimental setup for daytime imaging of the alpha source: (**a**) Alpha source, (**b**) Alpha camera, (**c**) Controlling laptop, (**d**) Power bank and supply.

**Figure 13:**
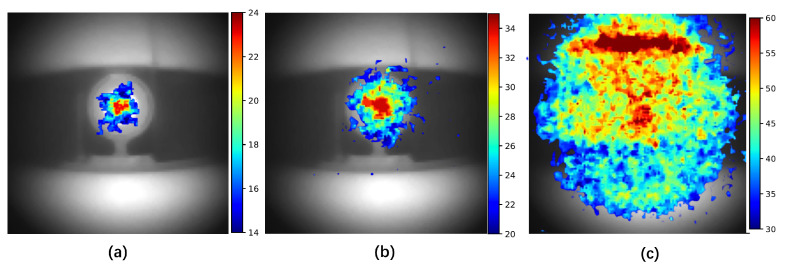
Experimental results for daytime UVC imaging of the alpha source at a 70 cm distance in 10 min. The alpha source signal is represented using a color map and overlaid on a visible light image of the alpha source. (**a**) Controlled group of detection in a dark room. (**b**) Under indirect sunlight, the alpha source signal is visible. (**c**) Under direct sunlight, the ambient background overwhelms the signal, making it undetectable.

Daytime experiments were conducted at Fenswood Farm, Bristol, UK, at 15:00 on 12 June 2024, under cloudy conditions. The setup for these experiments is illustrated in Figure 12. The camera was positioned to avoid directly facing toward the sun, capturing only indirect sunlight reflected from the ground and walls. The detection distance was 70 cm, and the exposure time was 10 min. This configuration allowed for the visualization of the alpha source signal with SNR of about 3, as shown in Figure 13b. Therefore, the lowest detectable activity under indirect sunlight would be $\frac{3\;\mathrm{MBq}}{3}=1\;\mathrm{MBq}$. To confirm that the detected signal originated from the alpha RL and not just from reflected sunlight, a controlled experiment was conducted in a dark room under similar settings, shown in Figure 13a. The similarity between the signal detected in a dark room and that under sunlight confirms the successful detection of alpha RL in sunlight conditions.

In contrast, when the camera faced direct sunlight, the intense ambient light overwhelmed the alpha source signal, making it indistinguishable, as shown in Figure 13b. Despite the use of a stack of six 276 nm short pass filters, the direct sunlight background could not be adequately reduced. This suggests that a small amount of sunlight below 280 nm might be reaching the ground, bypassing the filter range. Further research is needed to verify this hypothesis.

It is important to note that the use of stacked UVC filters is also effective for night-time detection, as the UV background at night is orders of magnitude lower than during the day. The preference for the 337 nm filter system at night stems primarily from the relatively low RL emission in the UVC region, which is 61 times lower than the signal in the 337 nm region. Additionally, the use of a stack of UVC filters diminishes the signal to just 3.1%. Thus, in conditions where background UV light is minimal, using the 337 nm filter system substantially enhances the signal. This strategy optimizes the detection capabilities of the system under various ambient light conditions.

It is also important to discuss the effect of gamma rays on the imaging system. Gamma rays emitted from Am-241 cannot be focused by the fused silica lens and will directly impact the camera sensor, resulting in intense peaks in individual pixels. As previously discussed, these effects can be mitigated using a median filter. As for the gamma-induced RL, according to the model proposed by Thompson et al. (2016) [27], the gamma rays from Am-241 are relatively weak and do not significantly contribute to RL emissions, making them a less useful parameter for RL emission considerations. Moreover, even for gamma background from strong gamma emitters like Co-60 and P-32, the intensity of gamma-induced RL, with activity levels similar to that of Am-241, is orders of magnitude lower compared with alpha-induced RL. This is because gamma rays can travel long distances, causing gamma-induced RL to be distributed over a much larger volume. Consequently, we can conclude that our imaging system is largely unaffected by gamma rays due to these factors.

## 4. Conclusions

This study successfully demonstrated the capability of long-range imaging of a relatively weak alpha-emitting radiation source through radioluminescence (RL) in an open environment under varying lighting conditions. A deep-cooled CCD camera was employed to accurately show the distribution of alpha RL. Additionally, a low f-number lens system designed to reduce the blue shift of filters was developed. Conducted at Fenswood Farm, Bristol, UK, the experiments provided clear insights into the challenges and capabilities of detecting alpha RL signals both during night-time and daytime conditions. This achievement is highly desirable for the nuclear industry, where such capabilities can significantly enhance safety and monitoring processes.

During night-time imaging, a sandwich filter system was employed to enhance the blocking ability. The filter centered at 337 nm captured the main emission of alpha RL, while a filter system centered at 343 nm facilitated background subtraction. The effectiveness of this filter system was confirmed as it successfully isolated the RL signal of a 3 MBq alpha source from ambient UV light at a 1 m detection distance within one minute. And the lowest detection limit can be calculated as 75 kBq. Different controlled artificial lighting sources were tested to simulate city light. Notably, the LED light source did not introduce additional UV background, pragmatically confirming that alpha imaging is viable within nuclear facilities when LED lighting is used. However, both incandescent and fluorescent lights posed challenges due to their inherent UV emissions, with the fluorescent lighting proving particularly problematic by overwhelming the alpha RL signal even after background subtraction.

During the daytime experiments, a stack of five tilted 276 nm short pass filters was used to reject sunlight background and capture alpha RL in the UVC region. This camera setup could detect a 3 MBq alpha source at 70 cm in 10 min under indirect sunlight. Direct sunlight, however, masked the alpha RL signal, emphasizing the need for careful planning of outdoor RL imaging applications.

These findings highlight the potential of RL imaging systems for environmental monitoring and scientific research in conditions that closely mimic real-world scenarios. To our knowledge, the alpha camera described in this paper is the most capable alpha RL imaging system ever demonstrated. The implications of this research extend into areas such as environmental monitoring, nuclear safety, and forensic science, where the accurate and efficient detection of radioactive materials under diverse environmental conditions is critical. The continued development and refinement of this technology promise to broaden its applicability and effectiveness in these vital fields.

## Figures and Tables

**Table 1:** Summary of optical filters used in the experiment. CWL: Center Wavelength, FWHM: Full Width at Half Maximum, OD: Optical Density.

Filter Name	Supplier	Specifications	Purpose
65-128	Edmund Optics, York, UK	Reflective filter, CWL: 337 nm, FWHM: 10 nm, OD: 4	Used to detect alpha RL at 337 nm.
39-343	Edmund Optics, York, UK	Reflective filter, CWL: 343 nm, FWHM: 5 nm, OD: 4	Employed to detect background radiation at 343 nm.
Hoya U340	UQG Ltd., Cambridge, UK	Absorptive filter, bandpass: 275–375 nm	Served to reduce multi-reflection between reflective filters.
FF01-276/SP-25	Laser 2000 Photonics, Cambridge, UK	Reflective filter, 276 nm short pass	Utilized to eliminate ambient sunlight interference.
FBH450-40	Thorlabs Ltd., Lancaster, UK	Reflective filter, CWL: 450 nm, FWHM: 40 nm, OD: 5	Captures images in the visible band to overlap with the alpha RL signal.

**Table 2:** Specifications of optical lenses used in the experiment. FL: Focal Length.

Lens Name	Supplier	Specifications
LA4372-UV	Thorlabs Ltd., Lancaster, UK	FL = 150.0 mm, Aperture = 75 mm
LC4513-UV	Thorlabs Ltd., Lancaster, UK	FL = −75.0 mm, Aperture = 25.4 mm
21-912	Edmund Optics, York, UK	FL = 25 mm, Aperture = 25 mm

**Table 3:** Specifications of artificial light sources used in the experiment.

Light Source	Supplier	Specifications
Fluorescent Light Tube	Megaman, Herts, UK	20 watts, 1151 lumen
Incandescent Light Bulb	Leuci, Buckinghamshire, UK	40 watts, 389 lumen
LED Light Bulb	Wilko, Bristol, UK	4.4 watts, 470 lumen

## Data Availability

Data are contained within the article.

## Abbreviations

The following abbreviations are used in this manuscript:

RL	Radioluminescence
PMT	Photomultiplier tube
CCD	Charge-coupled device
CWL	Center Wavelength
FWHM	Full Width at Half Maximum
OD	Optical Density
FL	Focal Length
EFL	Effective Focal Length
UV	Ultraviolet
